# Preoperative fibrinogen-to-albumin ratio, a potential prognostic factor for patients with stage IB-IIA cervical cancer

**DOI:** 10.1186/s12885-020-07191-8

**Published:** 2020-07-25

**Authors:** Qiang An, Wei Liu, Yujia Yang, Bing Yang

**Affiliations:** Department of Gynecology, Affiliated Hospital of Zunyi Medical University, No 149, Dalian road, Zunyi City, 563100 Guizhou Province China

**Keywords:** Cervical cancer, Prognosis, Fibrinogen-to-albumin ratio, Biomarker

## Abstract

**Background:**

Previous studies have shown that fibrinogen-to-albumin ratio (FAR) is a novel prognostic immune biomarker in various diseases. In this study, we investigated the role of FAR in the prognosis of patients with stage IB-IIA cervical cancer (CC).

**Methods:**

A total of 278 eligible participants with newly diagnosed CC (stage IB-IIA) who had undergone radical hysterectomy followed by adjuvant chemotherapy were enrolled in this study. Demographics, clinicopathological variables, and laboratory tests were obtained from the medical records. Risk factors for overall survival (OS) and recurrence-free survival (RFS) were evaluated by univariate and multivariate Cox proportional regression analyses. The association between OS, RFS, and FAR was assessed by the Kaplan–Meier method using log-rank test.

**Results:**

FAR was associated with age, International Federation of Gynecology and Obstetrics (FIGO) stage, depth of the invasion, and C-reactive protein (CRP) level (*P* < 0.05). Preoperative FAR was an effective predictor for OS in CC patients with a cut-off value of 7.75 and an area under the curve (AUC) of 0.707 (*P* < 0.001). The univariate and multivariate Cox analyses indicated that FIGO stage and FAR were two independent risk factors for both OS and RFS (*P* < 0.05). Kaplan–Meier analysis confirmed that patients with high FAR levels showed significantly lower RFS (*P* = 0.004) and OS (*P* = 0.003) than those with low FAR levels.

**Conclusions:**

This study indicated that elevated preoperative FAR might be a novel prognostic factor for CC patients with stage IB-IIA.

## Background

Cervical cancer (CC) is the most common cancer of the female genital tract and the fourth leading cause of malignancy-related deaths among women worldwide [1]. While the incidence and mortality of invasive CC have markedly decreased over time, it still ranks as the third most common cancer in women, with approximately 250,000 patients dying from CC each year globally [2]. Radical hysterectomy followed by chemotherapy or chemoradiation has been widely considered as the primary therapeutic strategy for locally advanced CC patients which is defined as International Federation of Gynecology and Obstetrics (FIGO) stage IB-IIA [3]. However, the prognosis is comparatively poor in patients with tumor recurrence due to the limited clinical therapies available [4]. To date, no well-established prognostic biomarkers for CC have been identified. Therefore, a non-invasive, easily accessible pre-treatment biomarker to predict tumor recurrence and prognosis in CC is urgently required.

Recently, the laboratory indexes including neutrophil-to-lymphocyte ratio (NLR) [5], prognostic nutritional index (PNI) [6], C-reactive protein (CRP)/albumin ratio (CAR) [7], and albumin-to-alkaline phosphatase ratio (AAPR) [8] have been validated as prognostic factors for CC. In recent decades, systemic inflammation status has served as an important hallmark of malignancy and is closely associated with the initiation, progression, metastasis, as well as resistance to drug therapy [9].

Albumin (Alb) and fibrinogen (Fib) are two commonly used circulating inflammatory proteins. Alb has been reported to be a well-established prognostic factor in patients with various diseases, including oral cavity cancer [10], metastatic pathological femur fractures [11], and amyotrophic lateral sclerosis [12]. Moreover, the prognostic role of Fib has also been reported in various studies, such as spontaneous intracerebral haemorrhage [13] and surgically resected non-small-cell lung cancer [14]. The Fib-to-Alb ratio (FAR), which considers both inflammatory biomarkers, is a novel prognostic immune biomarker in various diseases, e.g. gallbladder cancer [15], breast cancer [16], and ST-segment elevation myocardial infarction [17]. However, whether FAR could be a prognostic factor for CC has never been examined, which is the main aim of this study.

## Methods

### Patients

This single-center, retrospective study was performed with the approval of the Medical Ethics Committee of our hospital. Eligible participants with newly diagnosed CC admitted to Zunyi Medical University Affiliated Hospital, from June 2010 to December 2017 were enrolled in this study. The inclusion criteria were as follows: (a) newly diagnosed CC patients with the FIGO stage IB-IIA; (b) with clinicopathological and laboratory data including Alb and Fig; (c) who had undergone radical hysterectomy followed by adjuvant chemotherapy; (d) with five-year follow-up data. The exclusion criteria were as follows: (a) with the conditions affecting preoperative Alb or Fib expressions, e.g. infection, inflammation, hematological disease, autoimmune disease, abnormal liver or renal function; (b) combined with other malignancies; (c) without follow-up or complete data. Each enrolled participant was required to submit a signed informed consent.

### Treatment schedule

The diagnosis and treatment schedules of CC patients followed the guidelines by Bhatla et al. [18] All enrolled CC patients had undergone radical hysterectomy and bilateral pelvic lymphadenectomy. Based on the results of postoperative pathology, post-operative platinum-based adjuvant chemotherapy (two or three cycles) with or without concurrent radiotherapy was performed. The pathological definition was carried out by two independent experienced pathologists who were blinded to this study design.

### Data collection

The whole body computed tomography (CT) or positron emission tomography/computed tomography (PET/CT) examination was performed preoperatively. The demographics (including age, body mass index (BMI), and preoperative comorbidities) and clinicopathological variables (including pathological type, FIGO stage, tumor grade, maximum tumor size, adjuvant therapy, lympho-vascular space invasion (LVSI), lymphatic node metastasis (LNM), depth of invasion, and vaginal invasion were extracted from the medical records. Pre-operative laboratory parameters including blood cell analysis (e.g., white blood cell), blood biochemical analysis (e.g., Alb), coagulation analysis (e.g., Fib), and inflammatory cytokines (e.g., CRP) were routinely detected using the blood samples obtained 1 day prior to operation. As described in previous reports, the FAR was calculated by dividing Fib (mg/dL) by Alb (mg/dL), multiplied by 100 [16].

### Prognosis definition

The clinical and imaging examinations and laboratory tests were assessed for each visit during the follow up. The end point was set as overall survival (OS), recurrence-free survival (RFS), or the due date of follow-up (December 31, 2017). RFS was defined as the period from the initial surgery to tumor recurrence, death, or the due date. OS was defined as the period from the date of initial surgery to death or the due date.

### Statistical analysis

Statistical analysis was performed using GraphPad prism 8.0 and SPSS 19.0. The predictive and cut-off values of FAR for OS were assessed by receiver operating characteristic (ROC) curve using the Youden index. Chi-square test, Fisher’s exact test, Student t-test, or Mann–Whitney U test was performed as appropriate. Risk factors for OS and RFS were evaluated by univariate and multivariate analyses using Cox proportional models. OS and RFS analyses were carried out by Kaplan–Meier method using log-rank test. *P* < 0.05 was considered statistically significant.

## Results

### Patient characteristics

**Fig. 1:**
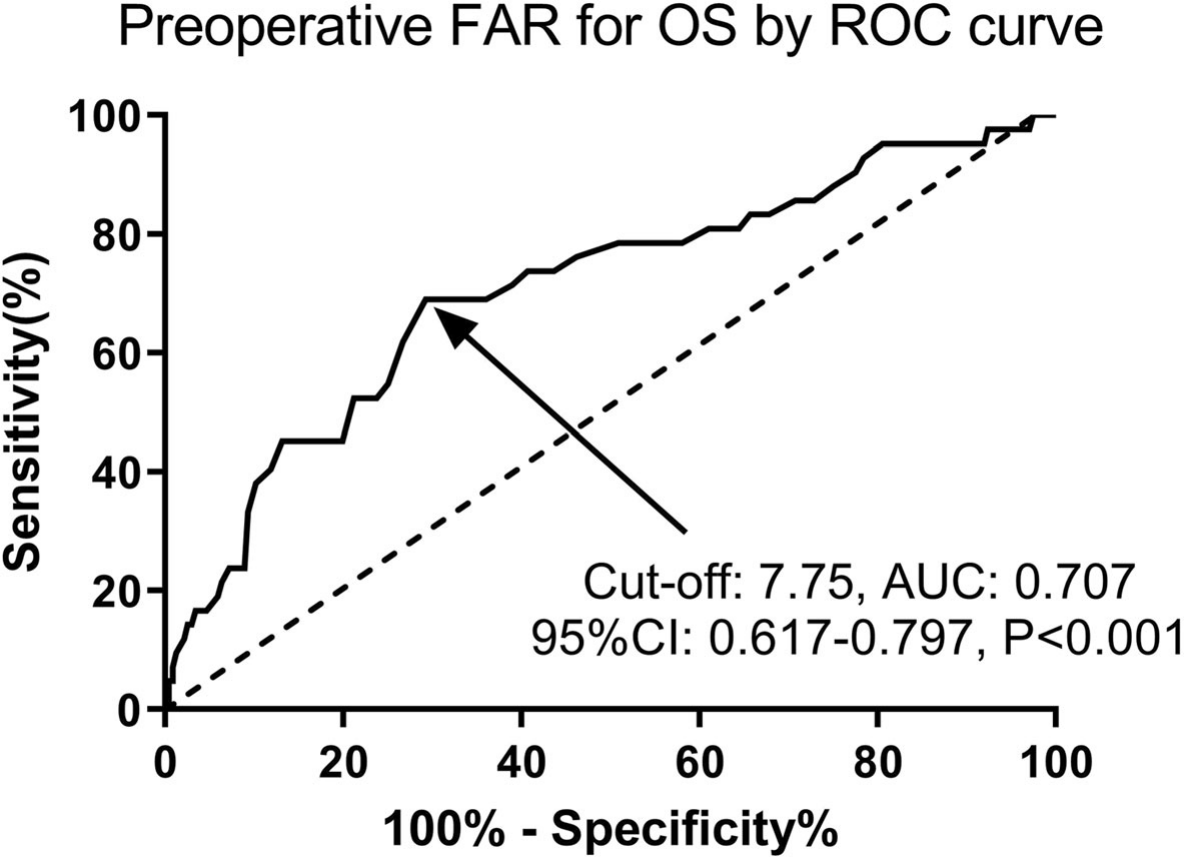
Predictive value of preoperative FAR for OS in CC patients (FIGO stage IB-IIA) by ROC curve. Preoperative FAR was an effective predictor for OS with an AUC of 0.707 and a cut-off value of 7.75 (sensitivity: 69.05%, specificity: 70.76%, *P* < 0.001). ROC, receiver operating characteristic; FAR, fibrinogen-to-albumin ratio; OS; overall survival; CC, cervical cancer; CI, confidence interval; AUC, the area under the curve

A total of 331 CC patients (stage IB-IIA) who had undergone radical hysterectomy were initially enrolled. Among these patients, 53 were excluded (13 with infection or inflammation, 6 with hematological disease, 7 with autoimmune disease, 8 with abnormal liver or renal function, and 19 missing follow-up or complete data) and 278 CC patients were included in the final analysis. According the ROC curve analysis, preoperative FAR was an effective predictor for OS in CC patients with a cut-off value of 7.75 and an area under the curve (AUC) of 0.707 (sensitivity: 69.05%, specificity: 70.76%, *P* < 0.001, see Fig. 1) using the Younden index method. Based on the cut-off values, enrolled patients were categorized into high FAR group (FAR> 7.75, *n* = 180, 64.7%) and low FAR group (FAR≤7.75, *n* = 98, 35.3%). The relationship between clinicopathological characteristics and FAR are listed in Table 1. The mean age of the whole cohort was 45.5 years and the majority, 89.2% (248/278), were with squamous cell carcinoma (SCC) patients. Patients with a low FAR were more prone to have a lower age (*P* = 0.016) and a lower FIGO stage (*P* = 0.033). Those CC patients with a low FAR had a low rate of depth of invasion over 2/3 (31.1% vs 44.9%, *P* = 0.022). No statistical difference was observed between high and low FAR groups with respect to BMI, preoperative comorbidities, pathological type, tumor grade, maximum tumor size, adjuvant therapy, LVSI, LNM, and vaginal invasion (*P* > 0.05). The laboratory tests associated with FAR in CC patients with stage IB-IIA are shown in Table 2. There was no significant difference between patients with high and low FAR except serum CRP concentration (*P* = 0.012).

**Table 1:** Clinicopathological variables associated with FAR in CC patients with stage IB-IIA

Parameters	Total	FAR≤7.75	FAR> 7.75	*p* value
Number	278	180	98	–
Age (years)	45.5 ± 6.3	44.8 ± 6.7	46.8 ± 6.4	0.016*
BMI (kg/m^2^)	22.3 ± 2.0	22.3 ± 2.1	22.4 ± 1.8	0.691
Comorbidities (n, %)				–
Diabetes	31 (11.2)	20 (11.1)	11 (11.2)	0.977
Hypertension	39 (14.0)	22 (12.2)	17 (17.3)	0.240
Hyperlipemia	26 (9.4)	17 (9.4)	9 (9.2)	0.943
Pathological type		–	–	0.297
SCC	248 (89.2)	158 (87.7)	90 (91.8)	–
Non-SCC	30 (10.8)	22 (12.3)	8 (8.2)	–
FIGO stage		–	–	0.033*
IB	146 (50.4)	103 (57.2)	43 (43.9)	–
IIA	132 (47.5)	77 (42.8)	55 (56.1)	–
Tumor grade (n, %)		–	–	0.590
G1	53 (19.1)	36 (20.0)	17 (17.3)	–
G2	166 (59.7)	109 (60.6)	57 (58.2)	–
G3	59 (21.2)	35 (19.4)	24 (24.5)	–
Maximum tumor size		–	–	0.447
≥ 4 cm	99 (35.6)	67 (37.2)	32 (32.7)	–
< 4 cm	179 (64.4)	113 (62.8)	66 (67.3)	–
Adjuvant therapy				0.748
No	54 (19.4)	42 (23.3)	12 (12.2)	–
Chemoradiotherapy	77 (27.7)	45 (25.0)	32 (32.7)	–
Chemotherapy	108 (38.8)	67 (37.2)	41 (41.8)	–
Radiotherapy	39 (10.8)	26 (14.4)	13 (7.2)	–
LVSI		–	–	0.409
No	247 (88.8)	162 (90.0)	85 (86.7)	–
Yes	31 (11.2)	18 (10.0)	13 (13.3)	–
LNM				0.547
No	232 (83.5)	152 (84.4)	80 (81.6)	–
Yes	46 (16.5)	28 (15.6)	18 (18.4)	–
Depth of invasion		–	–	0.022*
≥ 2/3	100 (36.0)	56 (31.1)	44 (44.9)	–
< 2/3	178 (64.0)	124 (68.9)	54 (55.1)	–
Vaginal invasion		–	–	0.738
No	260 (93.5)	169 (93.9)	91 (92.9)	–
Yes	18 (6.5)	11 (6.1)	7 (7.1)	–

*Abbreviations*: *CC* cervical cancer, *BMI* body mass index, *SCC* squamous cell carcinoma, *FIGO* International Federation of Gynecology and Obstetrics, *FAR* fibrinogen-to-albumin ratio, *LVSI* lympho-vascular space invasion, *LNM* lymphatic node metastasis

*P*-values were calculated by Student’s t test, Mann–Whitney U test or Chi-squared test

* *P* < 0.05

**Table 2:** Laboratory tests associated with FAR in CC patients with stage IB-IIA

Laboratory tests	Total	FAR≤7.75	FAR> 7.75	*p* value
Number	278	180	98	–
Hemoglobin (g/L)	104.9 ± 10.5	105.3 ± 10.2	104.3 ± 11.2	0.451
Platelet (10^9^/L)	171.9 ± 37.1	173.8 ± 40.5	168.5 ± 36.3	0.281
WBC(10^9^/L)	7.0 ± 1.9	7.0 ± 2.1	7.1 ± 1.7	0.686
Total cholesterol (mmol/L)	4.3 ± 0.8	4.3 ± 0.8	4.2 ± 0.7	0.300
CRP (mg/L)	6.1 ± 7.9	5.2 ± 6.9	7.7 ± 9.5	0.012*

*Abbreviations*: *WBC* white blood cell, *CRP* C-reactive protein, *CC* cervical cancer, *FAR* fibrinogen-to-albumin ratio

*P*-values were calculated by Student’s t test or Mann–Whitney U test

* *P* < 0.05

### Risk factors for RFS and OS

In the total cohort, the 3-year RFS and OS rates were 87.4% (243/278) and 89.9% (250/278), respectively. The 5-year RFS and OS rates were 79.5% (221/278) and 84.9% (236/278), respectively. To determine the potential risk factors for RFS and OS, univariate and multivariate Cox proportional regression analyses were performed. As illustrated by Table 3, FIGO stage (Hazard ratio (HR): 2.11, 95% confidence interval (CI): 1.14–3.79, *P* = 0.017), LNM (HR: 1.84, 95% CI: 1.12–2.88, *P* = 0.032) and preoperative FAR level (HR: 2.41, 95% CI: 1.36–4.11, *P* = 0.011) were three independent risk factors for RFS in CC patients with stage IB-IIA. In addition, FIGO stage (HR: 2.72, 95% CI: 1.18–5.01, *P* = 0.022) and preoperative FAR level (HR: 2.83, 95% CI: 1.41–5.35, *P* = 0.008) were two independent risk factors for OS in CC patients (Table 4).

**Table 3:** Risk factors for RFS in CC patients with stage IB-IIA by univariate and multiple Cox regression analysis

Variables	Univariate Multivariate			
	HR(95% CI)	*p* value	HR(95% CI)	*p* value
Age (high vs low)	1.47 (0.97–2.21)	0.076		
BMI (high vs low)	0.99 (0.67–1.48)	0.923		
Diabetes (yes vs no)	1.21 (0.82–1.77)	0.358		
Hypertension (yes vs no)	1.03 (0.63–1.65)	0.892		
Hyperlipemia (yes vs no)	1.11 (0.72–1.61)	0.628		
Pathological type (SCC vs non-SCC)	1.21 (0.30–4.93)	0.783		
FIGO stage (IIA vs IB)	2.33 (1.29–4.24)	0.009*	2.11 (1.14–3.79)	0.017*
Tumor grade (G2/3 vs G1)	1.16 (0.68–2.06)	0.553		
Tumor size (≥4 cm vs < 4 cm)	1.83 (1.26–4.27)	0.026*	1.57 (0.88–2.81)	0.136
Adjuvant therapy (yes vs no)	1.61 (0.47–5.11)	0.413		
LVSI (yes vs no)	1.09 (0.69–1.69)	0.676		
LNM (yes vs no)	1.59 (1.04–2.49)	0.029*	1.84 (1.12–2.88)	0.032*
Depth of invasion(≥2/3 vs < 2/3)	1.16 (0.46–2.92)	0.718		
Vaginal invasion (yes vs no)	1.24 (0.70–2.21)	0.411		
Hemoglobin (high vs low)	0.74 (0.31–1.79)	0.521		
Platelet (high vs low)	1.11 (0.60–2.04)	0.712		
WBC (high vs low)	1.66 (0.52–5.22)	0.388		
Total cholesterol (high vs low)	1.14 (0.73–1.82)	0.554		
CRP (high vs low)	1.32 (0.49–3.14)	0.533		
FAR (> 7.75 vs ≤7.75)	2.53 (1.24–5.18)	0.009*	2.41 (1.36–4.11)	0.011*

*Abbreviations*: *CC* cervical cancer, *BMI* body mass index, *SCC* squamous cell carcinoma, *FIGO* International Federation of Gynecology and Obstetrics, *FAR* fibrinogen-to-albumin ratio, *LVSI* lympho-vascular space invasion, *LNM* lymphatic node metastasis, *WBC* white blood cell, *CRP* C-reactive protein, *HR* hazard ratio, *CI* confidence interval

* *P* < 0.05

**Table 4:** Risk factors for OS in CC patients with stage IB-IIA by univariate and multiple Cox regression analysis

Variables	Univariate Multivariate			
	HR(95% CI)	*p* value	HR(95% CI)	*p* value
Age (high vs low)	1.74 (0.88–3.51)	0.113		
BMI (high vs low)	1.11 (0.56–2.14)	0.751		
Diabetes (yes vs no)	1.08 (0.58–2.02)	0.765		
Hypertension (yes vs no)	1.15 (0.61–2.11)	0.651		
Hyperlipemia (yes vs no)	1.43 (0.62–3.22)	0.357		
Pathological type (SCC vs non-SCC)	1.89 (0.92–4.11)	0.079		
FIGO stage (IIA vs IB)	2.91 (1.32–6.14)	0.008*	2.72 (1.18–5.01)	0.022*
Tumor grade (G2/3 vs G1)	0.98 (0.50–1.87)	0.914		
Tumor size (≥4 cm vs < 4 cm)	1.41 (0.56–3.31)	0.431		
Adjuvant therapy (yes vs no)	1.59 (0.93–2.68)	0.087		
LVSI (yes vs no)	2.53 (1.20–5.61)	0.016*	1.48 (0.81–2.69)	0.211
LNM (yes vs no)	1.84 (0.67–4.79)	0.211		
Depth of invasion(≥2/3 vs < 2/3)	1.41 (1.07–1.87)	0.018*	1.22 (0.83–1.77)	0.281
Vaginal invasion (yes vs no)	1.33 (0.96–1.78)	0.081		
Hemoglobin (high vs low)	0.70 (0.40–1.24)	0.265		
Platelet (high vs low)	1.55 (0.97–2.47)	0.061		
WBC (high vs low)	1.44 (0.95–2.11)	0.068		
Total cholesterol (high vs low)	1.26 (0.65–2.33)	0.443		
CRP (high vs low)	1.25 (0.84–1.81)	0.194		
FAR (> 7.75 vs ≤7.75)	2.78 (1.56–5.06)	0.003*	2.83 (1.41–5.35)	0.008*

*Abbreviations*: *CC* cervical cancer, *BMI* body mass index, *SCC* squamous cell carcinoma, *FIGO* International Federation of Gynecology and Obstetrics, *FAR* fibrinogen-to-albumin ratio, *LVSI* lympho-vascular space invasion, *LNM* lymphatic node metastasis, *WBC* white blood cell, *CRP* C-reactive protein, *HR* hazard ratio, *CI* confidence interval

* *P* < 0.05

### PFS and OS associated with FAR

**Fig. 2:**
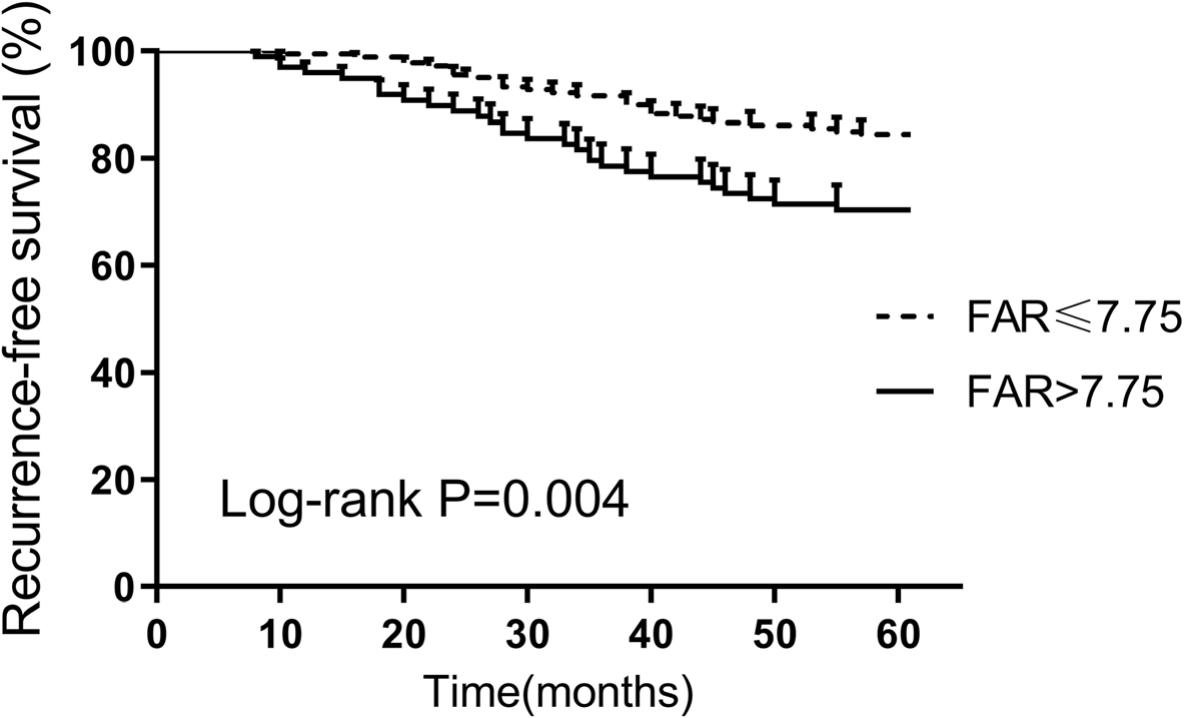
Recurrence-free survival in CC patients and preoperative FAR by Kaplan-Meier curve analysis. A higher preoperative FAR (> 7.75) correlated with a worse recurrence-free survival (*P* = 0.004). FAR, fibrinogen-to-albumin ratio; CC, cervical cancer

**Fig. 3:**
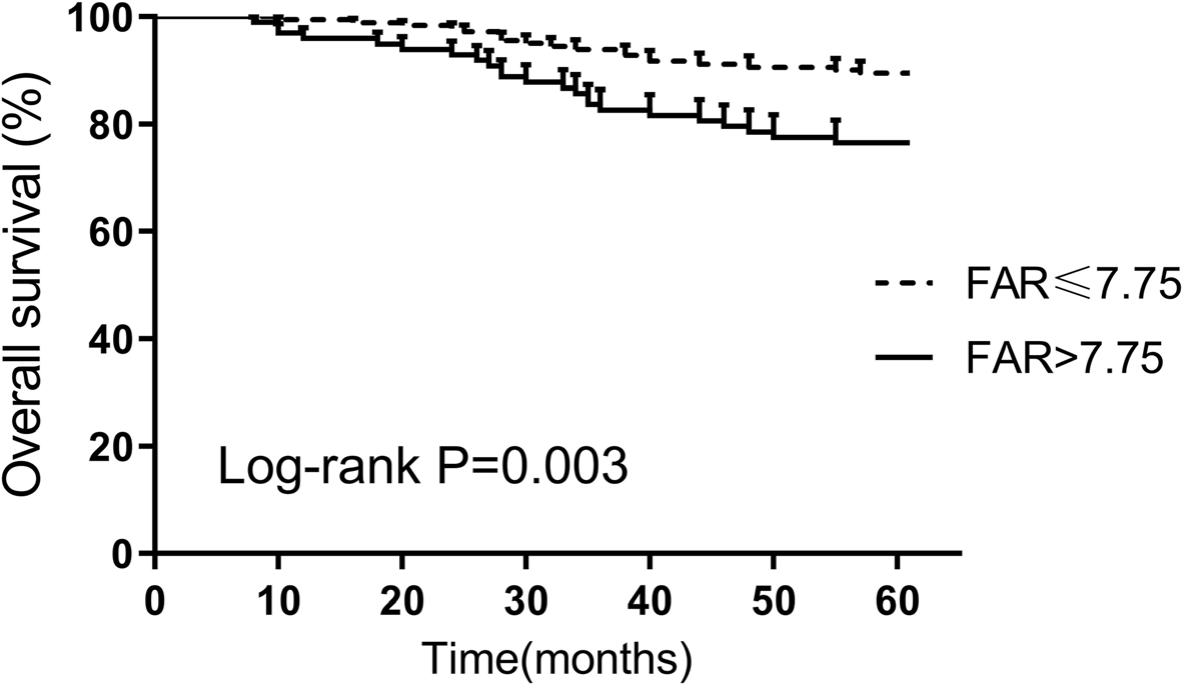
Overall survival in CC patients and preoperative FAR by Kaplan-Meier curve analysis. A higher preoperative FAR (> 7.75) correlated with a worse overall survival (*P* = 0.003). FAR, fibrinogen-to-albumin ratio; CC, cervical cancer

The results of the Kaplan–Meier analyses confirmed that patients with high FAR level showed significantly lower RFS and OS than those with low FAR by log-rank test (*P* = 0.004 in Fig. 2 and *P* = 0.003 in Fig. 3, respectively).

## Discussion

Cervical cancer is a significant threat to human health due to its high morbidity and mortality rate. Effective prognostic biomarkers may offer a better option for risk stratification and provide individual appropriate treatment strategies for CC patients. Parametrial involvement (PMI), positive surgical margins, and LNM are established prognostic factors for tumor recurrence, progression, and clinical outcomes [19]. However, these indexes require an accurate confirmation of postoperative pathology and are time consuming. Therefore investigation to identify an effective, simple, and economic prognostic biomarker for clinical outcomes in CC patients is needed.

In this study, we demonstrated that FAR was an independent prognostic indicator for CC patients with stage IB-IIA. To our knowledge, this was the first study concerning the prognostic implication of FAR in CC. Prognostic role of serum Alb and Fib has been reported in various studies. Taken together, the prognostic role of FAR was far superior to either Alb or Fib alone. In our study, FAR was observed to be associated with some clinicopathological indicators (i.e., age, FIGO stage, and depth of invasion), indicating a potential close correlation between FAR level and the aggressiveness and progression of CC. Fib is a key factor in the coagulation system [20], while Alb is an objective indicator reflecting the nutritional condition [21]. Increased Fib level is widely observed in patients with solid malignancies and is closely associated with the size, deep stromal invasion, progression, and recurrence of tumors [22, 23]. Meanwhile, there is increasing evidence that Alb may be used for the early diagnosis, prognosis, or prediction of solid malignancies [24]. A recent study by Seebacher et al. has indicated Alb as part of a prognostic model regarding recurrent CC [25].

Moreover, Fib and Alb are widely accepted as two acute phase response proteins for the systemic inflammatory status [26]. Fib and Alb are both synthesized by hepatocytes and they vary oppositely under inflammatory stimulation [27]. Taken together, FAR is a critical factor in nutrition condition, coagulation system, and systemic inflammation. Furthermore, these processes were all closely associated with the survival, intravasation, and adhesion of tumor cells, leading to increased metastatic potential [28], which might be a possible explanation for the prognostic role of FAR in CC patients. Furthermore, a recent study by Huang et al. has indicated that pretreatment Alb/Fib ratio could improve the diagnostic efficiency of CC alone or combined with tumor biomarkers [24].

The prognostic role of FAR has been reported for patients with malignancies in recent studies, including esophageal squamous cell carcinoma [29], breast cancer [16], metastatic colorectal cancer [30], hepatocellular carcinoma [31], and gallbladder cancer [15]. In addition, a recent study by Liu et al. indicates that FAR may act as a novel inflammatory parameter reflecting disease activity of ankylosing spondylitis [27]. The prognostic role of FAR is also observed in patients with non-ST elevation acute coronary syndrome after percutaneous coronary intervention [32]. A recent meta-analysis by Zhang et al. has uncovered the close correlation between FAR and positive lymph node metastasis, distant metastasis, deeper infiltration, and advanced clinical stage in human malignant tumors [33]. Similarly, a recent study by Yu et al. indicates preoperative albumin-to-fibrinogen ratio as an independent predictor for chemotherapy resistance and prognosis in advanced epithelial ovarian cancer [34]. All these studies are in line with our results. Furthermore, the optimal cut-off values of FAR for prognosis vary in different reports with unknown underlying mechanisms. The different biological behaviors of tumors, sample sizes, cohort characteristics, racial differences, and population heterogeneity might be potential explanations for the inconsistent findings. Therefore, more large-scale studies are urgently needed to verify the conclusions. However, we have to admit that the study findings and conclusions would be more robust if a validation cohort was used.

## Conclusions

In conclusion, this study indicated that elevated preoperative FAR might be a novel prognostic factor for CC patients with FIGO stage IB-IIA.

## Data Availability

Please contact the author Bing Yang (yangbing_zy@163.com) upon reasonable requests.

## Abbreviations

CC: Cervical cancer
SCC: Squamous cell carcinoma
BMI: Body mass index
FIGO: International Federation of Gynecology and Obstetrics
LVSI: Lympho-vascular space invasion
LNM: Lymphatic node metastasis
FAR: Fibrinogen-to-albumin ratio
WBC: White blood cell
CRP: C-reactive protein
RFS: Recurrence-free survival
OS: Overall survival
ROC: Receiver operating characteristic
AUC: Area under the curve
HR: Hazard ratio
CI: Confidence interval
